# Biologically active components in by‐products of food processing

**DOI:** 10.1002/fsn3.1665

**Published:** 2020-06-01

**Authors:** Yaseen Galali, Zagros A. Omar, S. Mohammad Sajadi

**Affiliations:** ^1^ Food Technology Department College of Agricultural Engineering Sciences Salahaddin University‐Erbil Erbil KRG‐Iraq; ^2^ Department of Nutrition Cihan University‐Erbil Erbil Iraq; ^3^ Department of Phytochemistry Scientific Research Centre Soran University Soran Iraq; ^4^ Department of Pharmacy Rwanduz Private Technical Institute Rwandus Iraq

**Keywords:** antioxidants, food by‐products, food processing, phytochemicals

## Abstract

Food by‐products happen at various stages of production and processing at home and on commercial scales. In the recent years, because of the fast‐growing food companies and production, food processing by‐products have gained a lot of interest and attracted many technical and health professionals as well as policy makers internally and internationally. Also, concerns are increasing about food by‐products due to their ecological and environmental impact on the planet. This is particularly of concern when large companies emit. Large quantities of food by‐products are thrown into environment in which they can be exploited technically, medicinally, and pharmaceutically. This is due to their chemical component and biologically active compounds of the by‐products. Therefore, this systematic review focuses on the food by‐product biological compounds present in different parts of the food products, particularly in some common foods such as fruits, vegetables, cereals, dairy products, meat, eggs, nuts, coffee, and tea. Moreover, the review also explains the kind of biologically active compounds and their quantity not just in edible foods, but also in part and types of the by‐product which then can be reused and recycled into different processes in order to extract and get benefit from.

## Graphical abstract

This review focuses on the food by‐products biological compounds present in different parts of the food products, particularly in some common foods such as fruits, vegetables, cereals, dairy products, meat, eggs, nuts, coffee and tea. Moreover, the review also explains the kind of biological active compounds and their quantity not just in edible foods, but also in part and types of the by‐product which then can be re‐used and recycled into different processed in order to extract and get benefit from.

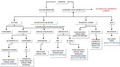

## 1 INTRODUCTION

There are rapidly growing scientific data and literature focusing on the role of food processing secondary and by‐products in relation to human well‐being. Coincidently, there is an increase in the consumers' information regarding noncommunicable diet‐associated diseases (Chernukhaa & Fedulova, 2015). Thus, the demand for nonchemical, natural, safe, and health‐improving food components is also growing (Schieber, Stintzing, & Carle, 2001). Large quantities of agricultural secondary or by‐products generated after food processing have become the main issue concerning food industry worldwide, since they could lead to environmental including pollution (soil, water, air). Furthermore, the disposal of agricultural by‐products could cost huge amount to treat under certain governmental regulations (Gowe, 2015). Additionally, it is no longer pragmatic to discard by‐products therefore; utilization of by‐products has become an alternative method to overcome this issue (Lafarga & Hayes, 2014; Zhao, Chen, & Du, 2012). Particularly, when food processing by‐products have considerable quantity of valuable and favorable raw bioactive functional compounds, therefore they can be useful for both technological and pharmaceutical purposes (Azyyati & Yen, 2014; Schieber et al., 2001).

For recovery of biologically active compound in food by‐products, different conventional methods including solvent‐based extraction have been used. However, due to more demand for eco‐friendly, cheap, and high‐efficiency methods, novel techniques are superior to conventional methods (Gençdağ, Görgüç, & Yılmaz, 2020) and novel methods have been studied and examined. The methods used include membrane‐based technologies including microfiltration, ultrafiltration, and nanofiltration (Castro‐Muñoz, Conidi, & Cassano, 2019), ultrasound‐assisted extraction (Sabater, Sabater, Olano, Montilla, & Corzo, 2020), microwave‐assisted extraction (Casazza, Pettinato, & Perego, 2020), electrotechnologies, ultrasound, high hydrostatic pressure, nanotechnology, and pressurized fluid (Zhu et al., 2020). The aim of this review was to present and give an overview about the most common biologically active compound present in a wide range of food products. Furthermore, the review also presents the by‐product bioactive compound in different food products such as milk, eggs, meat, cereals, fruit and vegetable, herbs and spices, coffee and tea, honey and sugar, and additives. Moreover, health and pharmaceutical benefits of the bioactive compounds will be presented (Table 1).

**TABLE 1:** Estimate of by‐products in the food industry

Industrial sector	Amount of waste (1,000 ton)	By‐products (%)
Production, processing, and preserving of meat and meat products	150	2.5
Production and preserving of fish and fish products	8	3.5
Production and preserving of fruits and vegetables	279	4.5
Manufacture of vegetable and animal oils and fats	73	1.5
Dairy products and ice cream industry	404	2
Production of grain and starch products	245	1.5
Manufacture of other food products	239	2
Drink industry	492	2
Total	1,890	2.6

## 2 METHODOLOGY

This systematic review was done through collecting data from databases such as PubMed and Web of Science as well as articles available in Google Scholar. The exclusion area included articles published before 1980, non‐peer‐reviewed articles, articles published in other languages than English, and nonoriginal articles including letters, editorial conference summaries, and paper without abstract. Therefore, only articles met these requirements were accepted.

## 3 FOOD PROCESSING BY‐PRODUCTS

### 3.1 Fruits

Fruits are one of the food materials that undergo industrial processing which could lead to various waste materials in different forms and shapes during pre‐ and postharvesting process in growing, preparation, and processing (Joshi & Devrajan, 2007). As a result of that diversity, the by‐product left is extremely dissimilar because of the difference in the fruits various industrial processes and producing different products. Some fruits result in 25%–30% by‐product or nonedible waste products (Ajila et al., 2009). However, less waste agribusiness has become a global need nowadays to overcome waste problem.

According to the fruit waste part; peel, seed, and stones produced after fruit processing, they could be importantly utilized as sources of many bioactive components for agroindustry to role in the aspect and transform these by‐products into valuable products (Tuchila, Jianu, Rujescu, & Butur, 2008). In a study about bioactive compounds, it was found that the mango peel and seed contained the highest quantity of bioactive compound. The peel included 5.997 mg of gallic acid/g of fresh weight (fw), 4.455 mg of quercetin/g fw, and 47.97% DPPH‐free radical scavenging activity at the concentration of 322 mg/ml, while the seed presented 37.279 mg of gallic acid/g, 35.954 mg of quercetin/g on fw basis, and 93.4% of DPPH‐free radical scavenging activity at the concentration of 307 mg/ml (Ayala‐Zavala, Rosas‐Domínguez, Vega‐Vega, & González‐Aguilar, 2010). In another study regarding the phenolic and antioxidant in some fruits including mango, longan, jackfruit, and avocado, following different analytical method ABTS (2,2‐azinobis‐3‐ethylbenzothiazoline‐6‐sulfonic acid), FRAP (ferric‐reducing antioxidant power), and FCR (Folin–Ciocalteu reagent) have been used. The research found that the seeds of these fruits contained more antioxidant capacity and phenolic content than the pulp. The ABTS, FRAP, and FCR values for the seeds of mango, longan, avocado, and jackfruit were as follows: 762, 448, 236.1, and 7.4 μmol of ascorbic acid/g; 2,572, 1,388, 1,484, and 2.8 μmol of gallic acid equivalents/g; and 117, 62.6, 88.6, and 27.2 mg of gallic acid equivalents/g, respectively. The ABTS, FRAP, and FCR values for the pulp of mango, longan, avocado, and jackfruit were as follows: 7.2, 3.7, 4.9, and 3.0 μmol of life‐protecting phytochemicals and antioxidants (Galali, Aziz, & Ali, 2017). Furthermore, depending on the type of the fruit and part of the fruit, high percentage of the antioxidants can be obtained such as vitamins C and E, phenolic compounds including phenyl‐propanoids and flavonoids, and/or carotenoids such as lycopene. Similar to the entire tissue, by‐products are rich in phytochemical, antioxidant, and antimicrobial compounds than the end products (Ayala‐Zavala et al., 2010). Therefore, recently the modern technology has focused on the utilization and exploitation of these by‐products in the production of new useful product with high technical and pharmaceutical properties as food additives and supplementation, since they possess many benefits which include antiviral, antibacterial, cardioprotective, and antimutagenic properties (Djilas, Canadanovic‐Brunet, & Cetkovic, 2009). Despite having this usefulness, there is no comprehensive utilization and exploitation due to lack of understating the pharmaceutical and economic benefits, so there is an important opportunity to ascorbic acid/ g: 36.6, 41.5, 9.6, and 6.8 μmol of gallic acid equivalents/g and 2.4, 1.6, 1.3, and 0.90 mg of gallic acid equivalents/g, respectively (Soong & Barlow, 2004). Another research noticed that the peel and seed of “Uba” mango had a total phenolic content of 0.0572 and 0.08254 mg/g on dry matter basis. These values are 4.6 and 7.3 times more than those in the pulp (Ribeiro, Barbosa, Queiroz, Knödler, & Schieber, 2008). It has been found that the phenolic compound of muscadine grapes in the seed was the highest followed by skin and pulp. Seventeen‐base units named isoprene. Terpenes or terpenoids are powerful against microbes. The peel and seed of avocado exhibited antimicrobial activity against some gram‐negative and gram‐positive bacteria and yeast. It was also observed that the seed and peel extract of “Hass” avocado showed a minimum inhibitory activity with 104.2 μg/ml against Salmonella enteriditis and Zygosaccharomyces bailii (Raymond Chia & Dykes, 2010). Lemon extracts were used in dairy products and improved the shelf life of mozzarella cheese. Pomegranate peel extracts showed antimicrobial activity against *Staphylococcus aureus* and *Bacillus cereus* in chicken and meat and increased shelf life by 3 weeks; compounds were identified in muscadine grapes. The compounds identified in seeds included hydrolyzable tannins, flavan‐3‐ols and condensed tannins, ellagic acid derivatives, and quercetin rhamnoside. The skin contained hydrolyzable tannins and flavonoids, including anthocyanin 3,5‐diglucosides, quercetin, myricetin, and kaempferol glycosides ( Sandhu & Gu, 2010). It has been found that the most common phenol compounds in grape seeds were seemed to be flavan‐3‐ols; most of them are gallocatechin gallate and catechin. The skins were mostly flavonols, that is, quercetin and myricetin. Determination of anthocyanins in the berry skin by ultra‐high‐performance liquid chromatography discovered twenty derivatives of malvidin, delphinidin, petunidin, cyanidin, and peonidin (Pantelić et al., 2016). Fruits by‐products are also important for antimicrobial activity against some pathogenic, spoilage bacteria, and yeasts. The most abundant studied antimicrobials are essential oils. Essential oils are natural and volatile having strong odor produced by plants (Bakkali, Averbeck, Averbeck, & Idaomar, 2008). One of them is terpenes. They composed of a combination of 5‐carbon and controlled rancidity during storage. Antibacterial activity of grapes extracts was studied. They showed positive activity against some pathogenic bacteria. Therefore, fruit by‐products are promising source of antimicrobial agents (Amaral, Ekins, Richards, & Knowles, 1998).

Fruit by‐products are also a good source of antioxidant that can be used as antibrowning agent that could happen to free cut fruits and reduced the quality of the products. It has been reported that low percentage of ascorbic acid reduced the browning reaction in fresh‐cut peaches and apple slices and fresh‐cut pineapple. Also, extracts from Palo Fierro rich in antioxidants reduced the browning of apple juice.

Fruit by‐products are a good source of natural colorant and pigments due to high stability, purity, availability, and low cost particularly when the synthetic colorants are publically rejected due to health concerns. One of the common colorants that is extracted from different fruit by‐products is grape pomace (Stintzing & Carle, 2004) blueberry(Bobinaitė et al., 2016) and some other guajiru, jambolao, jussara, and acai (Sousa De Brito et al., 2007).

Fruit by‐products are significantly rich in bioactive dietary fibers which are crucial in prevention in many diet‐associated diseases (Zhu, Du, Zheng, & Li, 2015). Dietary fiber and fiber‐rich by‐products of food processing present high technical and pharmaceutical properties. Thus, they have been supplemented to many food products (Elleuch, Bedigian, Roiseux, & Besbes, 2011). In a study about the percentage of neutral dietary fiber, acid dietary fiber cellulose, hemicelluloses, and lignin based on 100 g dry matter, it was found that the percentages of dietary fiber were up to 63% (Table 2; Verma & Joshi, 2000).

**TABLE 2:** Chemical composition of by‐products of various fruits

By‐products	Moisture	Protein	Fat	Minerals	Fiber	Carbohydrate
Apple pomace	‐	2.99	1.7	1.6	16.1	17.3
Mango seed kernel	8.2	8.5	8.8	3.6	‐	74.4
Jack fruit (inner and outer portions)	8.5	7.50	11.8	6.5	30.7	14.1
Jack fruit seeds	64.5	6.6	0.4	1.2	1.5	25.8
Jack seed flour	77	2.6	0.2	0.7	1.0	18.1
Passion fruit peel	81.9	2.5	0.1	1.4	5.0	‐
Banana peel	79.2	0.8	0.7	2.1	1.7	5.0
Sweet orange seeds	4.0	15.8	36.9	4.0	14.0	‐
Watermelon seeds	4.3	34.1	52.6	3.7	0.8	4.5
Watermelon seeds	6.8	21.0	33.0	4.0	30.0	‐
Pumpkin seeds	6.0	29.5	35.0	4.5	12.0	12.5
Banana central core	93.1	0.3	0.03	1.0	0.6	1.20
Outer hard fibrous sheath	91.9	0.1	0.06	0.98	1.8	2.4
Press juice from stem	98.6	0.05	‐	0.6	‐	0.4

Protein is also another component of the fruit by‐products particularly seed and kernel flour which can be an inexpensive source to be exploited for different purposes. Different fruits such as papaya, apple, watermelon, guava, orange, prickly pear, apricot, and paprika discovered to contain different quantity of various amino acids including leucine, isoleucine, methionine, phenylalanine, lysine, threonine, tyrosine, and valine. This adds additional nutrition value to the by‐products (Salem & Abd El‐Ghany, 2012).

### 3.2 Vegetables

Vegetable by‐products composed of different parts: peels, seeds, stones, and leaves. They could be source of different materials such as antioxidants such as vitamins C and E, phenolic compounds including phenyl‐propanoids and flavonoids, and/or carotenoids such as lycopene can be found (Ayala‐Zavala et al., 2010).

Vegetable by‐products are important sources of phenolic compounds that present and can be extracted using different solvents. Various by‐products seemed to contain different bioactive compounds. Asparagus waste seems to have the highest phenolic compounds, and tomato showed the lowest phenolic compounds (Table 3; Peschel et al., 2006). It was found that using acetone solvent could be very powerful to extract phenolic compounds. It has been reported that caffeic acid derivatives are the major component in artichoke by‐products with a prevalent range of caffeoylquinic acid derivatives with chlorogenic acid (5‐O‐caffeoylquinic acid) as the most important of these derivatives. Some other phenolic compounds such as the flavonoids apigenin and luteolin (both glucosides and rutinosides) as well as different cyanidin caffeoylglucoside derivatives were determined (Llorach, Espín, Tomás‐Barberán, & Ferreres, 2002). Furthermore, by‐product from lettuce showed the presence of hydroxycinnamic acids and flavonoids. The flavonoids made of flavones (luteolin derivatives) and flavonols (quercetin derivatives), whereas by‐products from chicory made of only of kaempferol derivatives (Llorach, Tomás‐Barberán, & Ferreres, 2004). Cauliflower by‐products also showed to contain phenolic compounds. It has been stated that cauliflower by‐products contain flavonoids and hydroxycinnamic acids (caffeic acid and sinapic acid). Flavonols such as kaempferol and quercetin with sinapic acid and glucose seemed to be the major phenolics available (Llorach, Espín, Tomás‐Barberán, & Ferreres, 2003).

**TABLE 3:** Total phenol content (mgGAE g^−1^) of vegetable by‐products using different solvents

Raw material	Water	Methanol	Ethanol	Acetone	Hexane
Artichoke	42.75 ± 12.17	95.65 ± 8.24	88.15 ± 4.99	102.33 ± 6.19	36.65 ± 5.87
Asparagus	89.40 ± 5.07	69.43 ± 7.06	60.14 ± 5.85	113.65 ± 17.73	29.33 ± 4.36
Tomato	12.15 ± 0.83	37.29 ± 2.08	42.00 ± 6.19	49.61 ± 9.52	30.24 ± 4.76
Broccoli	29.87 ± 1.58	25.58 ± 2.51	28.31 ± 1.69	36.18 ± 1.89	33.45 ± 2.32
Cucumber	18.41 ± 2.68	27.26 ± 1.80	16.96 ± 2.16	20.52 ± 2.59	26.71 ± 5.21
Endive	34.01 ± 6.79	17.18 ± 2.24	16.12 ± 2.48	23.66 ± 0.93	23.44 ± 2.46
Chicory	13.56 ± 1.81	25.51 ± 3.11	21.54 ± 3.58	14.16 ± 1.45	12.30 ± 1.80

Dietary fiber contributes to the major part of the discarded by‐products of vegetables. It has been studied that the total dietary fiber content in vegetable by‐products ranges from 40% to 82% including all the types of soluble and insoluble dietary fibers (Table 4; Goñi & Hervert‐Hernández, 2011). They impart a substantial quantity of biologically active compounds including polyphones and carotenoids associated with the fiber in the human digestive system. So, phytochemicals participate in the body well‐being through dietary fibers. Therefore, phytochemicals can be deemed as dietary fiber components in the perception of similarity in their resistance digestibility in the digestive tract. It can be seen that the by‐products are a rich sources of dietary fiber and other bioactive compounds and values can be added to it because of that (Saura‐Calixto & Serrano, 2007).

**TABLE 4:** Total dietary fiber content of different vegetable by‐products

By‐products	Quantity (100 g dry weight basis)
Cabbage outer leaves	40.5
Carob	53.0
Carrot	48
Cauliflower	65.0
Pepper	80.4
Peas, green‐frozen	82.3

Regarding the elements, there is dissimilar amount present in vegetable by‐products. But it can be concluded that fruit and vegetable wastes possess high level of organic material that can be utilized for different purposes (Asquer, Pistis, & Scano, 2013), particularly if they are used as a feed to livestock which could be a balanced diet in terms of micro‐, macro‐, and trace elements (Table 5).

**TABLE 5:** Elements in fruit by‐products

	Al mg/kg	As mg/kg	B mg/kg	Ba mg/kg	Ca mg/kg	Cd mg/kg	Co mg/kg	Cr mg/kg	Cu mg/kg	Fe mg/kg	Hg mg/kg	K mg/kg	Li mg/kg	Mg mg/kg	Mn mg/kg	Mo mg/kg	Na mg/kg	Ni mg/kg	Pb mg/kg	Se mg/kg	Sn mg/kg	Sr mg/kg	V mg/kg	Zn mg/kg
Apricot	0.9	<0.05	0.10	0.014	3.6	0.002	0.029	0.6	0.04	2.3	<0.05	29.8	<0.005	1.3	0.31	0.02	0.08	2.8	0.04	<0.1	<0.25	0.01	0.007	0.08
Banana	0.6	<0.05	0.06	0.005	1.7	<0.001	0.01	0.03	0.4	0.2	<0.05	51.7	<0.005	4.3	0.08	0.01	0.08	2.5	0.08	<0.1	<0.25	0.03	<0.002	0.4
Clementine	1.0	<0.05	0.13	0.014	16.5	<0.001	<0.008	0.005	0.9	0.1	<0.05	16.7	<0.005	2.1	0.02	0.003	0.2	1.2	0.1	<0.1	<0.25	0.1	<0.002	0.9
Lemon	3.1	<0.05	0.04	0.021	13.0	<0.001	<0.006	0.005	0.6	0.1	<0.05	19.7	<0.005	1.6	0.03	<0.001	0.4	0.4	0.3	<0.1	<0.25	0.07	<0.001	1.1
Melon	3.4	<0.05	0.03	0.001	3.70	<0.001	0.010	0.001	0.8	0.1	<0.05	37.6	<0.005	3.6	0.02	<0.001	1.1	0.5	0.1	<0.1	<0.25	0.02	<0.001	0.9
Orange	0.3	<0.05	0.05	0.016	17.	<0.001	0.01	0.02	0.05	0.2	<0.05	11.8	<0.005	1.6	0.03	0.007	0.1	2.4	0.02	<0.1	<0.25	0.23	<0.001	0.05
Peach	0.6	<0.05	0.04	<0.002	1.2	0.001	0.02	0.001	1.3	0.2	<0.05	19.0	<0.005	1.2	0.01	<0.002	0.1	3.2	1.50	<0.1	<0.25	0.02	<0.002	8.4
Pear	0.1	<0.05	0.06	0.001	0.9	<0.001	0.007	0.006	0.4	0.08	<0.05	7.8	<0.005	0.6	0.01	<0.002	0.2	0.9	0.3	<0.1	<0.25	0.01	<0.002	1.2
Pineapple	4.8	<0.05	0.02	0.009	3.0	<0.001	0.005	0.001	1.9	0.07	<0.05	12.9	<0.005	1.3	0.16	<0.001	0.08	0.5	0.05	<0.1	<0.25	0.02	<0.001	1.5
Watermelon	2.0	<0.05	0.03	0.006	1.5	0.001	0.01	0.2	0.004	0.8	<0.05	17.1	<0.005	1.6	0.16	0.007	0.08	2.2	0.01	<0.1	<0.25	0.01	0.002	0.02

Abbreviations: Al, aluminum; As, arsenic; B, boron; Ba, barium; Ca, calcium; Cd, cadmium; Co, cobalt; Cr, chromium; Cu, copper; Fe, iron; Hg, mercury; K, potassium; Li, lithium; Mg, magnesium; Mn, manganese; Mo, molybdenum; Na, sodium; Ni, nickel; Pb, lead; Se, selenium; Sn, Tin; Sr, strontium; V, vanadium; Zn, zinc.

Vegetable by‐products contain various chemical and bioactive compounds which can be used in different ways. Ash content was high in summer squash vines (23.3%), whereas the lowest percentage (4.8%) was found in potato (Table 6). Moreover, lowest organic matter was found in summer squash vines (77.8%) and the highest was found (94.8%) in baby corn husk. Snow pea protein showed the highest percentage of 23.2%. In addition, cellulose in pea vines and hemicellulose in baby corn husk were found with highest percentage by 36.8 and 32.1, respectively. As it is the source of table sugar, sugar beet leaves showed the highest percentage of sugar by 24.9. Regarding specific proteins, albumin in cauliflower leaves, globulin in cabbage leaves, prolamin in black chickpea plant, and glutelin in pea vines were found with 62.4, 16.2, 27.6, and 22.7, respectively. Finally, percentage of the phenolic content of the radish leaves seemed to be the highest by 6.9% comparing to others (Table 7; Wadhwa & Bakshi, 2013). The above data show that the vegetable by‐products are rich in bioactive compounds and can be useful pharmaceutically.

**TABLE 6:** Chemical composition and bioactive compounds in some vegetable by‐products

	Ash	Organic matter	Crude protein	Neutral detergent fiber	Neutral detergent solubles	Acid detergent fiber	Hemicellulose	Cellulose	Total sugar	Albumin	Globulin	Prolamin	Glutelin	Total phenol
Sugar beet leaves	21.0	78.9	21.9	42.3	57.8	21.1	21.2	11.4	24.9	60.6	12.7	12.0	14.7	2.9
Cauliflower leaves	13.7	86.4	17.0	27.5	72.5	19.4	8.1	15.2	18.6	62.4	12.9	9.1	15.6	5.9
Black chick pea plant	9.8	90.2	13.6	46.4	53.6	38.2	8.3	25.3	14.0	43.5	13.5	27.6	15.5	3.2
Cabbage leaves	15.8	84.2	19.9	33.7	66.3	22.6	11.1	13.7	20.6	54.3	16.2	8.2	21.3	5.9
Pea vines	10.0	89.9	11.8	60.0	40.0	49.9	10.0	36.8	6.4	56.9	12.4	7.9	22.7	4.5
Radish leaves	22.1	77.9	19.4	27.9	72.1	21.9	5.9	14.9	9.5	61.0	13.7	11.4	13.8	6.9
Summer squash vines	23.3	76.8	13.9	41.1	58.9	40.4	0.7	16.9	7.8	69.8	14.8	2.8	12.6	3.7
Baby corn husk	5.2	94.8	11.6	60.9	39.1	28.8	32.1	24.4	‐	‐	‐	‐	‐	‐
Carrot	8.2	91.8	9.9	9.0	91.0	8.0	1.0	7.0	‐	‐	‐	‐	‐	‐
Ensiled pea vines	9.0	91.0	13.1	59.0	41.0	49.0	10.0	34.0	‐	‐	‐	‐	‐	‐
Potato	4.8	95.2	9.5	‐	‐	‐	‐	‐	‐	‐	‐	‐	‐	‐
Snow peas	5.2	94.8	23.2	23.1	76.9	14.4	8.7	21.6	‐	‐	‐	‐	‐	‐
Sugar beet pulp	7.3	92.3	10.0	45.8	54.2	23.1	22.7	‐	‐	‐	‐	‐	‐	‐
Tomato pomace	6.0	94.0	22.1	63.0	37.0	51.0	12.0	12.0	‐	‐	‐	‐	‐	‐

**TABLE 7:** Elements in vegetable by‐products

	Al mg/kg	As mg/kg	B mg/kg	Ba mg/kg	Ca mg/kg	Cd mg/kg	Co mg/kg	Cr mg/kg	Cu mg/kg	Fe mg/kg	Hg mg/kg	K mg/kg	Li mg/kg	Mg mg/kg	Mn mg/kg	Mo mg/kg	Na mg/kg	Ni mg/kg	Pb mg/kg	Se mg/kg	Sn mg/kg	Sr mg/kg	V mg/kg	Zn mg/kg
Aubergin	3.4	<0.05	0.03	0.006	2.6	<0.001	<0.004	0.001	0.42	0.07	<0.05	20.7	<0.005	1.8	0.02	0.001	0.30	0.2	0.1	<0.1	<0.2	0.02	<0.001	0.49
Broccoli	0.07	<0.05	0.06	0.01	14.0	<0.001	<0.006	0.002	0.3	0.1	<0.05	27.46	<0.005	4.3	0.02	0.008	3.2	0.2	0.06	<0.1	<0.2	0.08	<0.001	0.3
Cabbage	0.09	<0.05	0.04	0.006	7.9	<0.001	0.004	0.001	0.1	0.08	<0.05	21.1	<0.005	3.0	0.02	0.002	3.4	0.5	0.04	<0.1	<0.2	0.04	<0.001	0.2
Cauliflower	0.1	<0.05	0.03	0.006	4.2	<0.001	0.005	0.000	0.3	0.08	<0.05	23.0	<0.005	2.8	0.02	0.003	2.8	0.7	0.03	<0.1	<0.2	0.02	<0.001	0.2
Clementine	1.0	<0.05	0.13	0.01	16.5	<0.001	<0.008	0.005	0.9	0.1	<0.05	16.7	<0.005	2.1	0.02	0.003	0.2	1.2	0.1	<0.1	<0.25	0.1	<0.002	0.9
Courgette	0.1	<0.05	0.02	0.002	2.3	<0.001	0.003	0.002	1.6	0.08	<0.05	18.5	<0.005	1.9	0.01	0.002	0.1	0.6	0.07	<0.1	<0.2	0.01	<0.001	1.1
Cucumber	2.2	<0.05	0.02	0.004	3.7	<0.001	<0.002	0.001	0.05	0.05	<0.05	12.4	<0.005	1.8	0.01	0.002	1.1	0.07	0.01	<0.1	<0.25	0.02	<0.001	0.06
Endive	1.0	<0.05	0.01	0.000	3.6	<0.001	0.002	0.025	0.08	0.1	<0.05	12.4	<0.005	1.3	0.03	0.001	0.1	0.3	0.02	<0.1	<0.25	0.00	<0.001	0.08
Fennel	1.7	<0.05	0.02	0.007	5.4	<0.001	0.018	0.080	0.35	0.5	<0.05	21.6	<0.005	1.3	0.09	0.005	5.5	0.3	0.1	<0.1	<0.2	0.03	0.001	0.5
Lemon	3.1	<0.05	0.04	0.02	13.5	<0.001	<0.006	0.005	0.69	0.10	<0.05	19.7	<0.005	1.6	0.03	<0.001	0.4	0.4	0.3	<0.1	<0.2	0.07	<0.001	1.1
Lettuce	3.0	<0.05	0.01	0.005	6.9	<0.001	0.026	0.038	0.3	0.4	<0.05	30.1	<0.005	1.6	0.08	0.001	1.2	0.2	0.1	<0.1	<0.25	0.02	<0.001	1.1
Onion	0.7	<0.05	0.07	0.02	9.2	<0.001	0.004	0.001	0.5	0.1	<0.05	12.2	<0.005	1.7	0.02	0.002	0.9	0.5	0.1	<0.1	<0.2	0.05	<0.001	0.8
Pepper	0.3	<0.05	0.01	0.001	0.6	<0.001	<0.004	<0.001	0.8	0.09	<0.05	15.8	<0.005	1.1	0.01	<0.001	0.1	0.77	0.09	<0.1	<0.2	0.00	<0.001	0.7
Potato	5.48	<0.05	0.07	0.017	4.8	<0.001	0.012	<0.001	0.4	0.1	<0.05	45.3	<0.005	3.3	0.03	<0.002	0.35	0.2	0.1	<0.1	<0.2	0.01	<0.002	0.7
Tomato	0.4	<0.05	0.01	0.000	1.2	<0.001	0.003	0.001	0.3	0.07	<0.05	9.9	<0.005	0.6	0.01	0.001	0.2	0.6	0.04	<0.1	<0.2	0.00	<0.001	0.3
Carrot	1.0	<0.05	0.03	0.021	6.2	<0.001	<0.004	0.004	0.1	0.1	<0.05	14.2	<0.005	2.2	0.03	0.003	8.7	0.1	0.02	<0.1	<0.25	0.03	<0.001	0.1

### 3.3 Coffee

A large quantity of by‐products are accumulated during the process of green bean coffee production which is estimated to be around 50% (Mussatto, Carneiro, Silva, Roberto, & Teixeira, 2010). There are different by‐products that are generated after green coffee bean production according to the method used. The by‐product of dry technique is primarily husk which includes the dried skin, pulp, and parchment by 0.18 ton per ton (Esquivel & Jiménez, 2012; Murthy & Madhava Naidu, 2012). The by‐product of wet technique is mainly coffee pulp and the coffee silver skin, and the last by‐product is spent coffee ground after brewing process.

The chemical composition of coffee by‐products of different studies from 2000 to 2009 is presented in Table 8. The total carbohydrate is between 35 and 72.3, and total fiber is ranged from 24 to 43. Protein content is ranged from 5 to 11, but the lowest content is mineral which is up to 10%.

**TABLE 8:** Bioactive compounds of coffee by‐products

Components	(Pandey et al., 2000)	(Brand et al., 2001)	(Ferraz & Silva, 2009)	(Gouvea, Torres, Franca, Oliveira, & Oliveira, 2009)	(Bekalo & Reinhardt, 2010)	(Shenoy et al., 2011)	(Murthy & Naidu, 2012)	(Murthy & Madhava Naidu, 2012)	(Srinivas Murthy, Navya, & Murthy Pushpa, 2013)
Total carbohydrate	57.8	35.0		72.3	‐	‐	‐	‐	‐
Total fiber	‐	30.8		‐	‐	‐	24 ± 5.9	43 ± 0.5	24.0
Hemicellulose	‐	‐	23.8	11.0	29.7	28.0	7.0 ± 3.0	‐	‐
Cellulose	‐	‐	23.1	16.0	24.5		43 ± 8.0	‐	43.0
Lignin	‐	‐	28.3	9.0	23.7	72.0	9.0 ± 1.6	‐	9.0
Pectin	12.4	‐	‐	‐	‐	‐	1.6 ± 1.2	‐	‐
Protein	9.2	5.2		7.0	‐	‐	8.0 ± 5.0	‐	11.0
Minerals	‐	10.7	‐	‐	‐	‐		‐	

Table 9 shows the procyanidin and flavonol content of coffee by‐product in Arabica and Robusta from Mexico, India, and China. The lowest amount of flavonols, 5 µg/g, was unraveled in Robusta from China. The Arabica coffee husk from Mexico has the highest amount of flavanols. The difference in the chemical components could be due to the bean green process and roasting degree (Mullen, Nemzer, Stalmach, Ali, & Combet, 2013).

**TABLE 9:** Procyanidins and flavanols in coffee by‐products in Arabica and Robusta from Mexico, India, and China

Procyanidin and flavonoids	Mexico		India		China	
	Arabica	Robusta	Arabica	Robusta	Arabica	Robusta
Quercetin‐O‐rutinoside	23.1 ± 9.9	8.1 ± 0.9	3.9 ± 2.2	6.4 ± 3.5	2 ± 0.5	0.7 ± 0.2
Quercetin‐3‐O‐rutinoside	153.8 ± 62.4	3.7 ± 0.2	1 ± 9.2	4.7 ± 2.5	9.8 ± 2.2	2.6 ± 0.5
Quercetin‐3‐O‐galactoside	1.4 ± 0.7	2.9 ± 0.3	0.8 ± 0.3	2.9 ± 1.7	0.2 ± 0	0.1 ± 0
(+)‐catechin	32.4 ± 7.8	n.d.	37.3 ± 15.6	7.7 ± 1.3	21.1 ± 6.2	0.1 ± 0.1
(−)epicatechin	17.9 ± 2.6	n.d.	4.6 ± 2.1	n.d.	16 ± 2.5	0.5 ± 0.5

Abbreviation: n.d., nondetected.

A number of studies have tried to use the coffee by‐product in different aspects for new products. Some researchers endeavored to produce bioethanol from the coffee by‐product. A combination of enzymatic treatment, heating, and steam was used. The results showed that ethanol efficiency of 83% per amount of glucose and yield bioethanol ranged from 0.426 ± 0.0015 g/L (Arrizon et al., 2012). Another study used high pressure and sulfuric acid to hydrolyze dried coffee by‐products. The results showed that ethanol yield of 82 g/kg dry coffee pulp and the amount of ethanol produced was 0.45 g/g sugar (Shenoy et al., 2011). Gouvea et al. (2009) optimized best condition for ethanol production; it was found that using *Saccharomyces Cerevisiae* in sticky coffee husk fermentation resulted in best yield condition with the optimal temperature at 30°C and 3 g yeast/L. The yielded bioethanol was 8.49 ± 0.29 g/100 g dry husk (13.57 ± 0.45 g ethanol/L).

For health benefits, coffee by‐products can be useful due to its content of bioactive compounds. Various studies have confirmed the fact that coffee by‐products could be useful for health purposes. In a study about the benefits of coffee by‐products in relation to prebiotic, antimicrobial, and antioxidant characteristics, it has been concluded that coffee silver skin and coffee spent grounds can potentially be used as functional ingredients. Furthermore, both of them can be useful as a source of prebiotic compounds, but melanoidins should be removed. Coffee silver skin and coffee spent grounds could be utilized as natural preservatives if used in large amount. Moreover, coffee silver skin and coffee spent grounds can be an important component to improve human health because of its antioxidant activity (Jiménez‐Zamora, 2015). In another study, the antimicrobial activity of biologically active in coffee waste was studied, and it was found that coffee by‐products showed an inhibitory activity against *S. aureus* and *Escherichia  coli*. A stronger inhibition was also observed against *Candida sp*. growth *(C. albicans*, *C. Krusei*, and *C. parapsilosis* [Sousa, Gabriel, Cerqueira, Manso, & Vinha, 2015]).

### 3.4 Tea

Tea is one of the most consumed drinks worldwide and possesses strong phenolic content (Vladić et al., 2016). One of the waste parts is stalk and stem which are rich in dietary fiber removed during tea processing. However, tea leave waste is the main by‐products of tea leaf industry. Tea by‐products are normally disposed of as compost, dumped into lands, and/or burned. But these are not a reasonable solution since they cause both environmental and economic problems (Hossain, Ko, & Yang, 2012).

It has been reported that the flavanols are the most important compound of tea polyphenols that predominantly include catechins, such as epicatechin, epicatechin gallate, epigallocatechin, epigallocatechin gallate, and catechin. Since the tea leftover could contain similar components but with different quantities, a study was done on phenolic content of the black tea waste sample, oven waste, and grade waste. The results showed that both contain similar amount of polyphenolic compounds (Table 10). It was expected that the waste material contained less bioactive compound comparing to the free tea (Güçlü Üstündağ et al., 2016). The study concluded that tea waste is an important source of antioxidant and antimicrobial activity that can be used for different purposes such as food, pharmaceutical, cosmetic, and agricultural sectors. It was also stated that aqueous ethanol solvents could be the cheapest, nontoxic green alternative for antioxidant and antimicrobial phenolics from tea waste.

**TABLE 10:** Phenolic compound of tea waste

Compounds	Phenolic content (mg/g DW)a	
	Oven waste	Grade waste
Catechins		
Epicatechin	n.d.	n.d.
Epigallocatechin	n.d.	n.d.
Catechin	n.d.	n.d.
Gallocatechin	4.6	3.9 ± 0.21b
Epigallocatechingallate	1.0	0.9 ± 0.08b
Gallocatechingallate	n.d.	n.d.
Epicatechingallate	0.2	0.31 ± 0.02a
Total catechins	6.01	5.21 ± 0.28b
Total theaflavins	16.0 ± 0.59b	11.5 ± 0.50c
Gallic acid	0.6	0.5 ± 0.03b
Caffeine	16.5 ± 0.50b	16.0 ± 1.29b

Abbreviation: n.d., nondetected.

Alongside with the phenolic compound, it has also been reported that there are a number of amino acids in the tea leaf. Glutamic acid is the highest with 9.8 g/100 g protein, and cysteine and methionine are the lowest with 1.4 g/100 g protein (Table 11).

**TABLE 11:** Amino acid composition of tea leaf waste

Amino acids	g/100 g protein
Alanine	4.80
Arginine	4.90
Aspartic acid	8.00
Cystine	1.4
Glutamic acid	9.8
Glycine	4.6
Histidine	2.5
Isoleucine	4.2
Leucine	7.4
Lysine	6.3
Methionine	1.4
Phenylalanine	4.3
Proline	4.2
Serine	4.3
Threonine	4.0
Tyrosine	3.3
Valine	5.0

Regarding micronutrients, it has been found that tea by‐products contain a number of elements as mentioned in Table 12 (Morikawa & Saigusa, 2008).

**TABLE 12:** The chemical composition of tea waste

Tea leaves waste	*C	*N	*CA	*K	*Mg	*Na	**Fe	**Zn	**M	**Cu
Quantity	502.9	502.9	6.7	13.1	30.4	6.6	187.	14.5	763.0	12.9

Abbreviations: *(mg/g);**(µg/g); C, carbon; CA, calcium; Cu, cupper; Fe; iron; Mg, magnesium; Mn, manganese; N, nitrogen; NA, sodium; Zn, zinc.

### 3.5 Dairy products

Dairy industry sector is the main and essential fraction of global food industry with having a magnificent quantity of watery waste. The most predominant waste that gained industrial attention is whey since it contains valuable bioactive nutrients.

Whey is one of the main by‐products of the cheese manufacture process which is about the watery by‐product left after the process. These by‐products contain many bioactive proteins such as *β*‐lactoglobulin, *α*‐lactalbumin, bovine serum albumin, and immunoglobulins (Galali & Hanee, 2019). It can be seen that the first two is particularly present in high concentration (Table 13). These perform important health functions. Therefore, this makes the whey valuable nutritionally (Asghar, Anjum, & Allen, 2011).

**TABLE 13:** The compounds of the by‐products of cheese

Compounds	Concentration (g/L)
α‐Lactoglobulin	1.5
B‐Lactoglobulin 3–4	3–4
Bovine serum, albumin	0.3–0.6
IgG, IgA, IgM	0.6–09
Lactoperoxidase	0.06
Lactoferrin	0.5

One of the main compounds of the milk by‐products and whey is bioactive peptides. They have been labeled as protein fraction which imparts positive influence on body well‐being through improving body functions. Recently, milk whey has gained attraction by the technicians and scientists to be used as a source of bioactive peptides for industrial purpose (Table 14). They can possess physiological benefits and contribute to the pharmaceutical and functional food formulation.

**TABLE 14:** Bioactive peptides hydrolyzed from the casein and whey (modified from Muro Urista, Álvarez Fernández, Riera Rodriguez, Arana Cuenca, & Téllez Jurado, 2011)

Peptides	Microorganisms/enzymes
b‐Lg, a‐La	Trypsin
Na‐Casein, b‐casein, B‐lactoglobulin, a‐lactoalbumin	Pepsin, trypsin, K‐proteinase, thermolysin LYQQP
as2‐Casein	Lactobacillus different
k‐Casein	Lactobacillus delbrueckii bulgaricus IFO13953
b‐lactoglobulin	Lactobacillus rhamnosus, pepsin, and corolase PP
b‐Casein	Lactobacillus bulgaricus
b‐Casein	Streptococcus thermophilus + � Lactococcus lactis biovar diacetylactis
b‐Casein	Proteinase from *Enterococcus faecalis*
a‐Casein	Trypsin
b‐Lg	Thermolysin
b‐Casein	Pepsin
Na‐casein	Alcalase
Na‐casein	Na‐casein enzyme culture of bacterium and plants
Na‐casein	Alcalase
Whey proteins	Alcalase
b‐Lg	N‐proteinase

### 3.6 Cereal products

It has been reported that the rice bran has the highest content of phytosterols by 4.5 mg/g bran. Furthermore, wheat germ and durum wheat, oat bran, and wheat bran had 2.4,1.8, 1.5, and 1.5 mg/g content of phytosterols, respectively, (Table 15; Jiang & Wang, 2005), whereas the lowest phytosterol found in oat hull was 0.7 mg/g. In another study, it has been found that oil yields (g kg^‒1^ dry weight) extracted from cereal waste products were as follows: 189 for rice bran*,* 112 for wheat germ*,* 74 for corn bran*,* 58 for oat bran*,* 41 for buckwheat bran*,* 39 for spelt bran*,* 33 for wheat bran, and 27 for rye bran. Furthermore, the major fatty acids determined in the samples were palmitic acid by 11.39%–17.23%, oleic acid by 11.76%–42.73%, linoleic acid by 35.54%–62.65%, and α‐linolenic acid by 1.05%–9.46%. Moreover, the total tocochromanols and phytosterols were quantified in the oils (0.369–3.763 and 1.19–35.24 g kg^−1^ of oil, respectively). The extracted oils from buckwheat and corn bran, and wheat germ were dominated by tocopherols (99.9%, 84.2%, and 96.5%, respectively), while the oat, rice, rye, spelt, and wheat bran oils seem to be rich in tocotrienols (73.9%, 79.6%, 78.1%, 90.6%, and 73.8%), respectively (Górnaś, Rudzińska, Raczyk, & Soliven, 2016).

**TABLE 15:** Total phytosterol contents (as mg free sterols/g lipids) in total lipid extracts of cereal by‐products

	Brassicasterol	Campesterol	Compestanol	Stigmasterol	Sitosterol	Sitostanol	Unknown 1	Unknown 2	Cycloartenol &like phytosterols	24‐Methylenecycloartanol‐&like phytosterols	Total mg/m lipids	Total mg/g Raw material
Rice bran	—	2.6	0.3	1.8	4.9	0.8	0.8	0.3	3.33^b^	5.25^c^	20.3	4.5
Wheat bran	0.5	3.6	1.7	0.2	4.5	2.4	0.6	0.3	0.48^d^	3.01^d^	17.6	1.2
Wheat germ	0.1	4.7	0.6	0.2	11.2	0.8	1.4	0.3	0.69^d^	1.03^e^	21.2	2.4
Durum wheat	0.5	2.1	2.9	0.3	4.7	2.1	1.1	0.1	0.31^d^	0.70^e^	15.0	1.8
Oat bran	—	0.2	0.04	0.1	1.5	0.07	0.6	0.0	0.26^b^	0.38^c^	3.4	1.5
Oat hull	—	0.6	0.09	0.5	4.0	0.4	0.7	0.20	0.53^b^	0.85^c^	8.1	0.7
Corn fine fiber	—	4.8	1.1	5.0	27.6	4.1	1.7	1.4	1.44^d^	0.82^e^	0.1	0.3

^a^ This unknown peak was identified as lanosterol in rice bran lipids and as avenasterol in wheat bran and oat bran lipids. It was not identified in other samples.

^b^ Identified as cycloartenol.

^c^ Identified as 24‐methylenecycloartanol.

^d^ Unidentified compounds that had the same GC retention time as cycloartenol but had different MS spectra.

^e^ Unidentified compounds that had the same GC retention time as 24‐methylenecycloartanol but had different MS spectra.

A dash (—) indicates not detected.

Another by‐product component in the cereal by‐product is dietary fibers in different quantities. Corn bran seems to be richest by‐product by 87.86 g followed by wheat bran 44.46 g and sesame coat by 42 g, whereas the lowest fiber content is in oat bran by 23.8 g (Table 16; Elleuch et al., 2011). These dietary fibers have been used in many products including breads (Al‐Dmoor & Galali, 2014; Galali, 2014).

**TABLE 16:** Dietary fiber content of some cereal by‐products (% on dry matter basis)

By‐product	Fiber quantity (g)
Rice bran	27.0
Wheat bran	44.4
Corn bran	87.8
Sesame coat	42.0
Oat bran	23.8

### 3.7 Nuts

Nut by‐products could include skin or testa, hard shell, green leafy cover, hull, and leaf. These are important and valuable sources of bioactive compounds that have multifunctional traits and antioxidant activity, and antimutagenic, anticarcinogenic, and antiproliferative properties (Shahidi & Ambigaipalan, 2015). The bioactive compounds of these components involve in the protection of the body directly or indirectly through detrimental free radicals and diminish the risks of the diet‐associated diseases. Thus, inclusion of these compounds in the daily meal is highly recommended by health expertise which can protect the body from harmful compounds (Alasalvar & Bolling, 2015). Regarding the nut coproducts, there are different biologically active compounds that have been determined and reported. Flavonoids including catechin, epicatechin, eriodictyol‐7‐O‐glucoside, quercetin‐3‐O‐rutinoside, quercetin‐3‐O‐galactoside, quercetin‐3‐O‐glucoside, kaempferol‐3‐O‐rutinoside, naringenin‐7‐O‐glucoside, isorhamnetin‐3‐O‐rutinoside, kaempferol‐3‐O‐glucoside, isorhamnetin‐3‐O‐glucoside, eriodictyol, quercetin, naringenin, kaempferol, and isorhamnetin are commonly found in the coproducts of almond, hazelnut, and pistachio. Recently, procyanidin dimers, trimers, and tetramers as well as dihydrochalcones such as phloretin‐2‐O‐glucoside have been reported only in hazelnut pellet. Isoflavones, such as daidzein and genistein, have not been found in pistachio coproducts compared with raw pistachio. Flavones, such as luteolin and 5, 7‐dihydroxychromone, were only found in peanut shell. In addition, apigenin is only determined in pistachio hard shell, while diosmetin is found only in peanut skin (Table 17). So far, no studies have been reported about the flavonoid contents of the shell and hull/ pellet of almond, peanut, and Brazil nut. In this connection, the flavonoids of cashew coproducts have not been reported here (Chang, Alasalvar, Bolling, & Shahidi, 2016).

**TABLE 17:** Bioactive compounds in nuts almond

Nuts	Bioactive compounds	Skin	Hard shell	Hull/pellet
Almond	Flavonols (g/g) Isorhamnetin Isorhamnetin‐3‐O‐glucoside Isorhamnetin‐3‐O‐rutinoside Kaempferol Dihydroxykaempferol Kaempferol‐3‐O‐glucoside Kaempferol‐3‐O‐rutinoside Dihydroquercetin Quercetin Quercetin‐3‐O‐glucoside Quercetin‐3‐O‐galactoside Quercetin‐3‐O‐rutinoside Flavan‐3‐ols (mg/100g) fresh weight (+)‐Catechin (−)‐Epicatechin (−)‐Epicatechin‐3‐gallate Unknown dimer A [(epi)catechin→ A→(epi)catechin] Unknown dimer A [(epi)catechin→A→(epi)catechin] Unknown dimer A [(epi)catechin→A→(epi)catechin] Unknown dimer A [(epi)catechin→ A→(epi)catechin] Flavanone (g/g) Naringenin Naringenin‐7‐O‐glucoside Eriodictyol Eriodictyol‐7‐O‐glucoside Anthocyanidins Procyanidin B3 + B1 Procyanidin B2 Procyanidin B3 Procyanidin B7 Procyanidin B5 Procyanidin C1 A‐type procyanidin dimer A‐type procyanidin dimer A‐type procyanidin dimer A‐type procyanidin dimer A‐type prodelphinidin dimer A‐type procyanidin trimer	4.19–4.87^a^, 10.9^b^ 8.9–15.6^a^, 139^b^27.6–41.4^a^, 639^b^1.71–1.96^a^, 2.4^b^49.9^b^ 1.65^a^, 23.2^b^ 12.8–31.8^a^, 196^b^ 0–10.3^a^ 1.43–1.78^a^, 3.12^b^ 1.33–2.41^a^ 6.45^b^ 8.15^b^ 20.1–38.3 7.2–26.5 0.15 2.4–3.5 1.2–4.8 3.2–4.9 2.5–4.9 83.4^a^ 6.84–22.1^c^ 2.75^a^ 0.8–1.6^c^ 11.8–23.8^c^ 5.34–16.1^c^ nd^c^ 5.63–13.9^c^ 3.5–8.6^c^ 3.45–15.3^c^ 3.2–7.0^c^ 1.4–6.3^c^ 4.0–7.3^c^ 0.7–2.04^c^ 0.9–1.8^c^ 1.6–4.3^c^	nr	
Brazil nut (g/g) defatted	Flavan‐3‐ols Catechin Gallocatechin Flavonols Quercetin Flavanonol Taxifolin	2875^d^ 1316^d^ 28.2^d^ 333^d^, 123^e^		
Hazelnut	Flavan‐3‐ols Catechin Epicatechin Epicatechin‐3‐gallate Procyanidin dimer 1 Procyanidin dimer 2 Procyanidin dimer 3 Procyanidin trimer 1 Procyanidin trimer 2 Procyanidin trimer 3 Procyanidin trimer 4 Procyanidin trimer 5	nr	0.3–0.8^f^	20.13^g^, ^h^ 9.26^g^ 1.37^g^ 99.2^g^ 19.2^g^ 0.4^g^ 7.0^g^ 2.47^g^ 14.7^g^ 8.4^g^ 3.7^g^
	Flavonoids` Procyanidin trimer 6 Procyanidin tetramer 1 Procyanidin tetramer 2 Procyanidin tetramer 3 Procyanidin B2 Flavonols Myricetin‐3‐O‐rhamnoside Quercetin‐pentoside Quercetin‐3‐O‐rhamnoside Quercetin‐3‐O‐rutinoside Hydrolyzable tannins B type dimer gallate Glansreginin A Glansreginin B Dihydrochalcones Phloretin‐2‐O‐glucoside	0.2^f^ 0.2–1.0^f^		3.61^g^ 2.37^g^ 2.3^g^ 6.83^g^ nr^g^ 17.7^g^ nr 50.14^g^ nr 0.97^g^ 39.26^g^ 71.71^g^ 6.53^g^ 18.7^g^
Pecan	Flavan‐3‐ols mg/g dry weight Catechin Epicatechin Epigallocatechin g/ml Epicatechin gallate		nr 102^i^, 0.3^j^ 120^k^,1326^l^ 0.3^k^,0.9	
Peanuts	p‐Coumaroyl‐O‐pentosid (mg/100g dw) Flavonols Isorhamnetin Quercetin Flavone Diosmetin Stilbenes trans‐Resveratrol Proanthocyanidins Proanthocyanidin A‐type dimers Proanthocyanidin B‐type dimers Proanthocyanidin trimers Proanthocyanidin tetramers Proanthocyanidin hexamers Proanthocyanidin heptamers Proanthocyanidin octamers Flavanone (mg/g) dry weight Eriodictyol Flavone Luteolin 5,7‐Dihydroxychromone	5.5 1.5 2.1 0.4 0.3 6.2 0.7 21.1 19.5 8.3 15.4 13.6 6.9	0.9 2.4 0.5	
Pistachio	Flavan‐3‐ols g/g extract Catechin Epicatechin Flavanols Procyanidin dimer Flavanone Hesperidin Eriodictyol‐7‐O‐glucoside Eriodictyol‐3‐O‐hexoside Eriodictyol Naringenin Naringenin‐7‐O‐neohesperidoside Flavone Apigenin Luteolin Flavonols Quercetin Quercetin‐3‐O‐rutinoside Quercetin‐3‐O‐glucoside Quercetin‐3‐O‐hexoside Kaempferol Myricetin Isoflavones Daidzein Genistein Genistein‐7‐O‐glucoside Anthocyanins Cyanidin‐3‐O‐galactoside Cyanidin‐3‐O‐glucoside	377, 140 105, 27.5 nr, 55 nr 366 0.21–3.35 63.2, 14 11.4, 2 119 nr^m^ nr, 30 17.8^m^, 13.7^n^ 5.1 49 2.68 0.9 1.6 Nr nr nr 5865^m^, 21.1^n^ 32.6^m^, 0.6^o^	nr–1240 630–980 nr 95–120 nr nr nr nr nr 145–190 nr 255–620 nr nr nr nr	
Walnut	Flavan‐3‐ols mg/kg fw Catechin Procyanidin dimer 1 Procyanidin dimer 2 Procyanidin trimer Procyanidin tetramer Hydrolysable tannins Galloyl bis HHDP glucose 1 Galloyl bis HHDP glucose 2 Glansreginin B Glansreginin A HHDP digalloyl glucose isomer 1 HHDP digalloyl glucose isomer 2 HHDP digalloyl glucose isomer 3 HHDP galloyl glucose 1 HHDP galloyl glucose 2 Di‐galloylglucose Di‐HHDP glucose isomer 1 Di‐HHDP glucose isomer 2 Di‐HHDP glucose isomer 3 Di‐HHDP glucose isomer 4 Vescalagin isomer 1 Vescalagin isomer 2 Vescalagin isomer 3 Vescalagin isomer 4 Vescalagin isomer 5 Vescalagin isomer 6 Flavonols Q‐galloyl pentoside 1 Q‐galloyl pentoside 2 Q‐galloyl pentoside Unknown 429 Unknown 459			26.9^p^ 157^p^ 1.2^p^ 1.8^p^ 281^p^ 95.5^p^ 50.9^p^ 22^p^ 597^p^ 35.5^p^ 47.5^p^ 14.53^p^ 22^p^ 57.5^p^ 21.3^p^ 115^p^ 133^p^ 43.4^p^ 27.8 32.4^p^ 49.4^p^ 22.7 100^p^ 35.3^p^ 17.1^p^ 4.89^p^ 1.4^p^ 4.8^p^ 16^p^ 2.9^p^

Abbreviation: Nr, none detected.

^a^ Data are expressed as g/g skin.

^b^ Data are expressed as g/g skin.

^c^ Range (minimum – maximum) values (expressed in g/100 g dw) obtained from eight varieties.

^d^ Bound phenolics.

^e^ Free phenolics.

^f^ Range (minimum – maximum) values (free phenolics expressed as g/g dw of kernel or hard shell.

^g^ Values (mg/kg).

^h^ Values obtained from dry‐blanched.

^i^ Shell extract.

^j^ Extract of whole shell.

^k^ Ethanolic extract.

^l^ Extract treated with infusion and spray dryer.

^m^ Bound phenolic acids.

^n^ Mean content obtained from six Argentinian varieties.

^o^ Values (expressed in g/g dw).

^p^ Values (expressed in g/g fw).

### 3.8 Egg

Egg by‐products mainly include eggshells and membranes (King'ori, 2017), wastewater from egg processing industry (Xu, Sheldon, Larick, & Carawan, 2002), and outcomes from breaking facilities and unsellable eggs (El‐Deek, Al‐Harthi, & Attia, 2011). Eggshells are waste products that are generated by different sources such as home, hatcheries, and fast food industry and which causes environmental pollution. The disposal challenges include eggshells include cost, availability of disposal places, smell, flies, and abrasiveness. However, they can be utilized for plants, human, and animal nutrition and produce collagen (Amu, Fajobi, & Oke, 2005).

Eggshell has been reported possessing many important nutrients such a calcium and trace amounts of other micro‐elements, that is, magnesium, boron, copper, iron, manganese, molybdenum, sulfur, silicon, and zinc (King'ori, 2017). A medium‐sized complete eggshell could give 750–800 mg of calcium. The calcium with magnesium and vitamin D increases mineral bone density (Schaafsma et al., 2018). Eggshells are also used as a cheap source of calcium in fertilizing plants (Amu et al., 2005). Shell membrane is an important by‐product source for collagen which can be used medicinally and industrially (Ogawa, Portier, Moody, & Bell, 2004). Also, eggshell and membrane contain many biologically active peptides (Table 18; Nakano, Ikawa, & Ozimek, 2003).

**TABLE 18:** Amino acid composition of decalcified eggshell and eggshell membranes (Mol%)

Amino acids	Decalcified eggshell	Inner shell membrane	Outer shell membrane
Asx	8.1	8.4	8.8
Thr	6.2	6.9	6.9
Ser	9.7	9.2	9.2
Glx	11.8	11.1	11.9
Gly	13.0	11.1	10.6
Ala	6.9	4.6	4.1
Val	7.3	7.2	7.9
Met	2.0	2.3	2.3
Ile	2.6	3.3	3.4
Leu	6.1	5.6	4.8
Tyr	1.8	2.2	1.7
Phe	2.1	1.6	1.5
His	4.2	4.1	4.3
Lys	3.6	3.6	3.4
Arg	5.9	5.7	5.8
Pro	8.3	11.6	12.0
Hyp1	0.3	1.5	1.4

Abbreviations: Ala, alanine; Arg, arginine; Asx, asparagine; Glx, glutamate; Gly, glycine; His, histidine; Hyp, hydroxyproline; Ile, isoleucine; Leu, leucine; Lys, lysine; Met, methionine; Phe, phenylalanine; Pro, proline; Ser, serine; Thr, threonine; Tyr, tyrosine; Val, valine.

Wastewater from egg industry is another by‐product of egg that contains many biologically active peptides. It can be seen that there are a number of peptides in the wastewater which some of them are destroyed with acid hydrolysis (Table 19; Xu et al., 2002).

**TABLE 19:** Amino acid composition of egg by‐products (wastewater) (g amino acid/100 g total protein)

Amino acids	Electrocoagulation
Threonine	4.1
Valine	6.1
Cystine^a^	—
Methionine^a^	—
Isoleucine	5.6
Leucine	8.4
Phenylalanine	5.1
Tryptophan^a^	—
Lysine	6.4

^a^ Destroyed by acid hydrolysis.

Protein peptides are also other bioactive compounds that are produced from egg processing by‐products. In a study about the egg‐yolk protein by‐product as a source of biologically active compounds, the following peptides sequences are produced: RASDPLLSV, RNDDLNYIQ, LAPSLPGKPKPD, and AGTTCLFTPLALPYDYSH. Peptides have pharmaceutical, medicinal, and functional properties in foods (Eckert, Zambrowicz, Pokora, & Setner, 2014).

### 3.9 Meat

**FIGURE 1:**
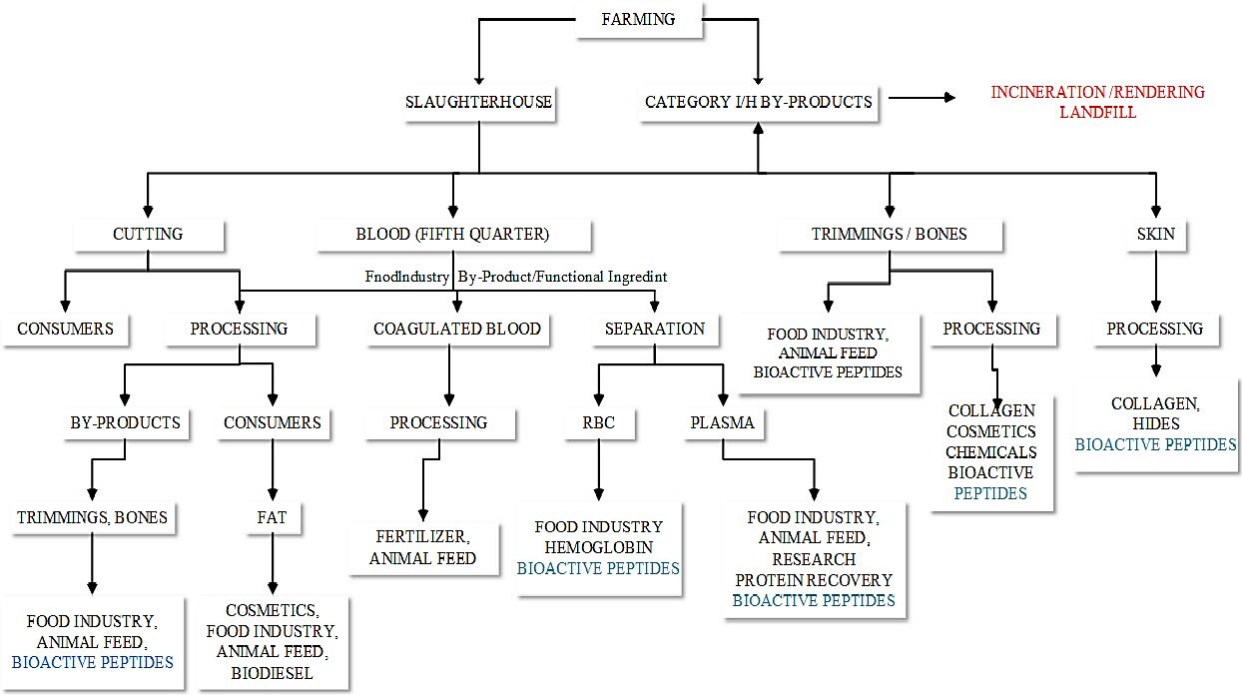
Major utilizes of meat muscle and by‐products

Meat like any other food products has a number of waste parts, but it is dissimilar depending on traditions, culture, and religion. However, general meat processing by‐products include skin, bones, meat trimmings, blood, fatty tissues, horns, feet, hoofs, or internal organs (Toldrá, Aristoy, Mora, & Reig, 2012). Meat by‐products are rich in many nutrients such as carbohydrate, protein, and fat that have different applications (Figure 1). Furthermore, collagen is one of the most abundant bioactive compounds of skin, bone, and horn (Gómez‐Guillén, Giménez, & López‐Caballero, 2011). Despite having medicinal and industrial usage, collagen is a precursor for many bioactive peptides with the sequences of antihypertensive, antithrombotic, and dipeptidyl peptidase‐IV (DPP‐IV, EC 3.4.14.5; Minkiewicz, Dziuba, & Michalska, 2011).

Blood is another by‐product of meat which is rich in protein such as fibrinogen, globulins, and albumins and hemoglobin (Bah, Bekhit, Carne, & Mcconnell, 2013). Blood and other by‐products are sources of bioactive peptides with different biological activities (Table 20; Lafarga & Hayes, 2014).

**TABLE 20:** Bioactive peptides in meat and meat by‐products with some modification

Bioactivity	Source	Parental protein
PEP‐inhibitory	Bovine brain	38–55 glial fibrillary acidic
Opioid	Bovine blood	Hemoglobin
Opioid	Bovine blood	Hemoglobin
Antithrombotic	Porcine muscle	‐
Antithrombotic	Porcine muscle	‐
Antioxidant	Porcine muscle	Integrin α‐3
Antioxidant	Porcine muscle	Collagen α‐1 (VII)
Antioxidant	Venison muscle	‐
Antioxidant	Venison muscle	‐
Antioxidant	Porcine muscle	Actin
Antioxidant	Porcine muscle	‐
Antioxidant	Porcine muscle	Tropomyosin
Antioxidant	Porcine muscle	Tropomyosin
Antioxidant	Porcine muscle	Myosin heavy chain
Antioxidant	Porcine muscle	‐
Antioxidant	Buffalo horn	‐
Antioxidant	Buffalo horn	‐
Antioxidant	Buffalo horn	‐
Antioxidant	Bovine muscle	1–12 myoglobin
Antioxidant	Bovine muscle	1–13 myoglobin
Antioxidant	Porcine blood	Hemoglobin
Antioxidant	Skin	Collagen
Antioxidant	Porcine blood	Plasma globulin/albumin
Antioxidant	Porcine blood	Plasma proteins
Antioxidant	Porcine blood	Plasma proteins
Antimicrobial	Bovine blood	33–61 α‐hemoglobin
Antimicrobial	Bovine blood	1–23 α‐hemoglobin
Antimicrobial	Bovine blood	107–136 α‐hemoglobin
Antimicrobial	Bovine blood	107–141 α‐hemoglobin
Antimicrobial	Bovine blood	137–141 α‐hemoglobin
Antimicrobial	Bovine blood	133–141 α‐hemoglobin
Antimicrobial	Bovine blood	126–145 β‐hemoglobin
Antimicrobial	Bovine blood	α‐Hemoglobin
Antimicrobial	Beef muscle	‐
Antimicrobial	Bovine blood	Hemoglobin
PEP‐inhibitory	Bovine brain	38–55 glial fibrillary acidic
Opioid	Bovine blood	Hemoglobin
Opioid	Bovine blood	Hemoglobin
Antithrombotic	Porcine muscle	‐

Animal horn is another waste product with many nutrients that can act and possess biologically active compound. In a study about elemental analysis of animal horn, there were a number of important elements including P, K, Ca, Mn, Fe, and Zn. The study also analyzed elemental composition of bone. It was found that there are same elements as horn plus Cr, Cd, Sn, and Ag (Buddhachat, Klinhom, & Siengdee, 2016).

## 4 CONCLUSIONS

To summarize, food processing by‐products accumulate in tons and cause huge environmental and economic problems in different stage and food industry sectors including fruits, vegetables, cereals, meat, dairy products, eggs coffee, and tea. If this would be studied and exploited carefully, they can be recycled and reused in different areas such as food industry, pharmaceuticals, and other biotechnical areas. These food by‐products are still containing many important biologically active compounds including fatty acids, amino acids, vitamins, minerals, dietary fibers, and antioxidants which can be useful economically and pharmaceutically (e.g., antimicrobials) in different aforementioned sectors instead of throwing into the ground. It is worth mentioning that different novel techniques have been examined in order to increase the potent of recovery of bioactive compounds from food by‐products. They are superior to conventional methods. This attributes the fact that they are eco‐friendlier, less hazardous, and less expensive. Therefore, it is important to choose a method that suits the intended bioactive compound to be extracted from different parts of the by‐product.

## CONFLICT OF INTEREST

The authors declare that they do not have any conflict of interest.

## ETHICAL APPROVAL

This study does not involve any human or animal testing.

## References

[fsn31665-bib-0001] Ajila, C. M. , Aalami, M. , Krishnarau, L. , Aalami, M. , Leelavathi, K. , & Prasada Rao, U. J. S. (2009). Mango peel powder: A potential source of antioxidant and dietary fiber in macaroni preparations Author's personal copy Mango peel powder: A potential source of antioxidant and dietary fiber in macaroni preparations. Innovative Food Science and Emerging Technologies, 11(1), 219–224. 10.1016/j.ifset.2009.10.004

[fsn31665-bib-0002] Alasalvar, C. , & Bolling, B. W. (2015). Review of nut phytochemicals, fat‐soluble bioactives, antioxidant components and health effects. British Journal of Nutrition, 113(S2), S68–S78. 10.1017/S0007114514003729 26148924

[fsn31665-bib-0003] Al‐Dmoor, H. , & Galali, Y. (2014). Novelty formulas of free gluten flat bread for celiac disease patients. World Journal of Medical Sciences, 11(3), 306–311.

[fsn31665-bib-0004] Amaral, J. A. , Ekins, A. , Richards, S. R. , & Knowles, R. (1998). Effect of selected monoterpenes on methane oxidation, denitrification, and aerobic metabolism by bacteria in pure culture. Applied and Environmental Microbiology, 64 520–525. 9464387 10.1128/aem.64.2.520-525.1998 PMC106076

[fsn31665-bib-0005] Amu, O. , Fajobi, A. , & Oke, B. O. (2005). Effect of eggshell powder on the stabilizing potential of lime on an expansive clay soil. Journal of Applied Science, 5(8), 1474–1478.

[fsn31665-bib-0006] Arrizon, J. , Mateos, J. C. , Sandoval, G. , Aguilar, B. , Solis, J. , & Aguilar, M. G. (2012). Bioethanol and xylitol production from different lignocellulosic hydrolysates by sequential fermentation Nutrición materno‐infantil View project Enzymatic fructosylation of natural compounds View project. Article in Journal of Food Process Engineering, 35(3), 437–454. 10.1111/j.1745-4530.2010.00599.x

[fsn31665-bib-0007] Asghar, A. , Anjum, F. M. , & Allen, J. C. (2011). Utilization of dairy byproduct proteins, surfactants, and enzymes in Frozen Dough. Critical Reviews in Food Science and Nutrition, 51(4), 374–382. 10.1080/10408391003605482 21432700

[fsn31665-bib-0008] Asquer, C. , Pistis, A. , & Scano, E. A. (2013). Characterization of fruit and vegetable wastes as a single substrate for the anaerobic digestion. Environmental Engineering and Management Journal, 12(S11), 89–92.

[fsn31665-bib-0009] Ayala‐Zavala, J. F. , Rosas‐Domínguez, C. , Vega‐Vega, V. , & González‐Aguilar, G. A. (2010). Antioxidant enrichment and antimicrobial protection of fresh‐cut fruits using their own byproducts: Looking for integral exploitation. Journal of Food Science, 75(8), R175–R181. 10.1111/j.1750-3841.2010.01792.x 21535513 PMC3032914

[fsn31665-bib-0010] Azyyati, S. N. , & Yen, G. B. (2014). Screening of antioxidant potential from cereal wastes and fruit peels. International Journal of Engineering Research & Technology, 3(1), 1990–1997.

[fsn31665-bib-0011] Bah, C. S. F. , Bekhit, A. E. D. A. , Carne, A. , & Mcconnell, M. A. (2013). Slaughterhouse blood: An emerging source of bioactive compounds. Comprehensive Reviews in Food Science and Food Safety, 12(3), 314–331. 10.1111/1541-4337.12013

[fsn31665-bib-0012] Bakkali, F. , Averbeck, S. , Averbeck, D. , & Idaomar, M. (2008). Biological effects of essential oils – A review. Food and Chemical Toxicology, 46, 446–475. 10.1016/j.fct.2007.09.106 17996351

[fsn31665-bib-0013] Bekalo, S. A. , & Reinhardt, H. W. (2010). Fibers of coffee husk and hulls for the production of particleboard. Materials and Structures/Materiaux et Constructions, 43(8), 1049–1060. 10.1617/s11527-009-9565-0

[fsn31665-bib-0014] Bobinaitė, R. , Pataro, G. , Raudonis, R. , Vškelis, P. , Bobinas, Č. , Šatkauskas, S. , & Ferrari, G. (2016). Improving the extraction of juice and anthocyanin compounds from blueberry fruits and their by‐products by pulsed electric fields. IFMBE Proceedings, 53, 363–366. 10.1007/978-981-287-817-5_80

[fsn31665-bib-0015] Brand, D. , Pandey, A. , Rodriguez‐Leon, J. , Sevastianos, R. , Brand, I. , & Soccol, C. R. (2001). Packed bed column fermenter and kinetic modeling for upgrading the nutritional quality of coffee husk in solid‐state fermentation. Biotechnology Progress, 17(6), 1065–1070. 11735442 10.1021/bp010112+

[fsn31665-bib-0016] Buddhachat, K. , Klinhom, S. , & Siengdee, J. B. (2016). Elemental analysis of bone, teeth, horn and antler in different animal species using non‐invasive handheld X‐ray fluorescence. PLoS One, 11(5), 1–21. 10.1371/journal.pone.0155458 PMC4873253 27196603

[fsn31665-bib-0017] Casazza, A. A. , Pettinato, M. , & Perego, P. (2020). Polyphenols from apple skins: A study on microwave‐assisted extraction optimization and exhausted solid characterization. Separation and Purification Technology, 240, 116640 10.1016/j.seppur.2020.116640

[fsn31665-bib-0018] Castro‐Muñoz, R. , Conidi, C. , & Cassano, A. (2019). Membrane‐based technologies for meeting the recovery of biologically active compounds from foods and their by‐products. Critical Reviews in Food Science and Nutrition, 59, 2927–2948. 10.1080/10408398.2018.1478796 29787307

[fsn31665-bib-0019] Chang, S. , Alasalvar, C. , Bolling, B. , & Shahidi, F. (2016). Nuts and their co‐products: The impact of processing (roasting) on phenolics, bioavailability, and health benefits–A comprehensive review. Journal of Functional Foods, 26, 88–122.

[fsn31665-bib-0020] Chernukhaa, I. M. , Fedulova, L. V. , & Kotenkova, E. A. (2015). Meat by‐product is a source of tissue‐specific bioactive proteins and peptides against cardio‐vascular diseases. Procedia Food Science, 5, 50–53. 10.1016/j.profoo.2015.09.013

[fsn31665-bib-0021] Ciemniewska‐Żytkiewicz, H. , Verardo, V. , Pasini, F. , & Bryś, J. (2015). Determination of lipid and phenolic fraction in two hazelnut (*Corylus avellana* L.) cultivars grown in Poland. Food Chemistry, 168, 615–622. 25172755 10.1016/j.foodchem.2014.07.107

[fsn31665-bib-0022] Djilas, S. , Canadanovic‐Brunet, J. , & Cetkovic, G. (2009). By‐products of fruits processing as a source of phytochemicals. Chemical Industry and Chemical Engineering Quarterly, 15(4), 191–202. 10.2298/CICEQ0904191D

[fsn31665-bib-0023] Eckert, E. , Zambrowicz, A. , Pokora, M. , & Setner, B. (2014). Egg‐yolk protein by‐product as a source of ACE‐inhibitory peptides obtained with using unconventional proteinase from Asian pumpkin (*Cucurbita ficifolia*). Journal of Proteomics, 110, 107–116. 25138009 10.1016/j.jprot.2014.08.003

[fsn31665-bib-0024] El‐Deek, A. , Al‐Harthi, M. , & Attia, Y. (2011). Effect of different dietary levels of dried eggs by‐product without or with shell on the performance of laying strain chicks from 2 to 8 wk of age. Archiv Für Geflügelkunde, 75, 20–29.

[fsn31665-bib-0025] Elleuch, M. , Bedigian, D. , Roiseux, O. , & Besbes, S. (2011). Dietary fibre and fibre‐rich by‐products of food processing: Characterisation, technological functionality and commercial applications: A review. Food Chemistry, 124, 411–421.

[fsn31665-bib-0026] Esquivel, P. , & Jiménez, V. M. (2012). Functional properties of coffee and coffee by‐products. Food Research International, 46, 488–495. 10.1016/j.foodres.2011.05.028

[fsn31665-bib-0027] Fabani, M. P. , Luna, L. , Baroni, M. V. , Monferran, M. V. , Ighani, M. , Tapia, A. , … Feresin, G. E. (2013). Pistachio (*Pistacia vera* var Kerman) from Argentinean cultivars. A natural product with potential to improve human health. Journal of Functional Foods, 5(3), 1347–1356. 10.1016/j.jff.2013.05.002

[fsn31665-bib-0028] Ferraz, F. D. O. , & Silva, S. S. D. (2009). Characterization of coffee husk biomass for biotechnological purposes. New Biotechnology, 25, S256.

[fsn31665-bib-0029] Galali, Y. (2014). Quality and shelf life of Pita and Tandoori breads supplemented with three novel functional ingredients. Plymouth, UK: University of Plymouth‐UK.

[fsn31665-bib-0030] Galali, Y. , Aziz, K. I. , & Ali, S. (2017). The antimicrobial activity of peel and seeds extracts of red grapes. Journal of Tikrit University for Agriculture Sciences, 17(3), 36–40.

[fsn31665-bib-0031] Galali, Y. , & Hanee, A. (2019). Miraculous properties of camel milk and perspective of modern science. Journal of Family Medicine and Disease Prevention, 5(1), 1–7.

[fsn31665-bib-0032] Gençdağ, E. , Görgüç, A. , & Yılmaz, F. M. (2020). Recent advances in the recovery techniques of plant‐based proteins from agro‐industrial by‐products. Food Reviews International. 10.1080/87559129.2019.1709203

[fsn31665-bib-0033] Gómez‐Guillén, M. , Giménez, B. , & López‐Caballero, M. (2011). Functional and bioactive properties of collagen and gelatin from alternative sources: A review. Food Hydrocolloids, 25, 1813–1827.

[fsn31665-bib-0034] Goñi, I. , & Hervert‐Hernández, D. (2011). By‐products from plant foods are sources of dietary fibre and antioxidants. London, UK: INTECH Open Access.

[fsn31665-bib-0035] Górnaś, P. , Rudzińska, M. , Raczyk, M. , & Soliven, A. (2016). Lipophilic bioactive compounds in the oils recovered from cereal by‐products. Journal of the Science of Food and Agriculture, 96(9), 3256–3265. 10.1002/jsfa.7511 26522347

[fsn31665-bib-0058] Gouvea, B. M. , Torres, C. , Franca, A. S. , Oliveira, L. S. , & Oliveira, E. S. (2009). Feasibility of ethanol production from coffee husks. Biotechnology Letters, 31(9), 1315–1319. 10.1007/s10529-009-0023-4 19466561

[fsn31665-bib-0036] Gowe, C. (2015). Review on potential use of fruit and vegetables by‐products as a valuable source of natural food additives. Food Science and Quality Management, 45, 47–61.

[fsn31665-bib-0037] Güçlü Üstündağ, Ö. , Erşan, S. , Özcan, E. , Özan, G. , Kayra, N. , & Ekinci, F. Y. (2016). Black tea processing waste as a source of antioxidant and antimicrobial phenolic compounds. European Food Research and Technology, 242(9), 1523–1532. 10.1007/s00217-016-2653-9

[fsn31665-bib-0038] Hossain, M. E. , Ko, S. Y. , & Yang, C. J. (2012). Dietary supplementation of green tea by‐products on growth performance, meat quality, blood parameters and immunity in finishing pigs. Journal of Medicinal Plants Research, 6, 2458–2467.

[fsn31665-bib-0039] Jiang, Y. , & Wang, T. (2005). Phytosterols in cereal by‐products. JAOCS, Journal of the American Oil Chemists' Society, 82(6), 439–444. 10.1007/s11746-005-1090-5

[fsn31665-bib-0040] Jiménez‐Zamora, A. S. P. (2015). Revalorization of coffee by‐products. Prebiotic, antimicrobial and antioxidant properties. Food Science and Technology, 61, 12–18.

[fsn31665-bib-0041] John, J. , & Shahidi, F. (2010). Phenolic compounds and antioxidant activity of Brazil nut (*Bertholletia excelsa*). Journal of Functional Foods, 2, 196–209.

[fsn31665-bib-0042] Joshi, V. , & Devrajan, A. (2007). Natural product radiance materials and methods. Natural Product Radiance, 7(2), 127–132.

[fsn31665-bib-0043] King'ori, A. M. (2017). A Review of the uses of poultry eggshells and shell membranes INDIGENOUS CHICKEN LAYERS View project. International Journal of Poultry Science, 10(1), 908–912. Retrieved from https://www.researchgate.net/publication/279557572

[fsn31665-bib-0044] Lafarga, T. , & Hayes, M. (2014). Bioactive peptides from meat muscle and by‐products: Generation, functionality and application as functional ingredients. Meat Science, 98, 227–239. 10.1016/j.meatsci.2014.05.036 24971811

[fsn31665-bib-0045] Llorach, R. , Espín, J. C. , Tomás‐Barberán, F. A. , & Ferreres, F. (2002). Artichoke (*Cynara scolymus* L.) Byproducts as a potential source of health‐promoting antioxidant phenolics. Journal of Agricultural and Food Chemistry, 50(12), 3458–3464. 10.1021/jf0200570 12033811

[fsn31665-bib-0046] Llorach, R. , Espín, J. C. , Tomás‐Barberán, F. A. , & Ferreres, F. (2003). Valorization of cauliflower (*Brassica oleracea* L. var. botrytis) by‐products as a source of antioxidant phenolics. Journal of Agricultural and Food Chemistry, 51(8), 2181–2187. 10.1021/jf021056a 12670153

[fsn31665-bib-0047] Llorach, R. , Tomás‐Barberán, F. A. , & Ferreres, F. (2004). Lettuce and chicory byproducts as a source of antioxidant phenolic extracts. Journal of Agricultural and Food Chemistry, 52(16), 5109–5116. 10.1021/jf040055a 15291483

[fsn31665-bib-0048] Ma, Y. , Kosińska‐Cagnazzo, A. , Kerr, W. L. , Amarowicz, R. , Swanson, R. B. , & Pegg, R. B. (2014). Separation and characterization of phenolic compounds from dry‐blanched peanut skins by liquid chromatography–electrospray ionization mass spectrometry. Journal of Chromatography A, 1354, 64–81. 10.1016/j.chroma.2014.06.027 25016324

[fsn31665-bib-0049] Minkiewicz, P. , Dziuba, J. , & Michalska, J. (2011). Bovine meat proteins as potential precursors of biologically active peptides‐a computational study based on the BIOPEP database. Food Science and Technology International, 17, 39–45. 21364044 10.1177/1082013210368461

[fsn31665-bib-0050] Morikawa, C. K. , & Saigusa, M. (2008). Recycling coffee and tea wastes to increase plant available Fe in alkaline soils. Plant and Soil, 304(1–2), 249–255. 10.1007/s11104-008-9544-1

[fsn31665-bib-0051] Mullen, W. , Nemzer, B. , Stalmach, A. , Ali, S. , & Combet, E. (2013). Polyphenolic and hydroxycinnamate contents of whole coffee fruits from China, India, and Mexico. Journal of Agricultural and Food Chemistry, 61(22), 5298–5309. 10.1021/jf4003126 23650984

[fsn31665-bib-0052] Muro Urista, C. , Álvarez Fernández, R. , Riera Rodriguez, F. , Arana Cuenca, A. , & Téllez Jurado, A. (2011). Review: Production and functionality of active peptides from milk. Food Science and Technology International, 17, 293–317. 10.1177/1082013211398801 21917640

[fsn31665-bib-0053] Murthy, P. S. , & Madhava Naidu, M. (2012). Sustainable management of coffee industry by‐products and value addition—A review. Resources, Conservation and Recycling, 66, 45–58. 10.1016/j.resconrec.2012.06.005

[fsn31665-bib-0054] Murthy, P. S. , & Naidu, M. M. (2012). Recovery of phenolic antioxidants and functional compounds from coffee industry by‐products. Food and Bioprocess Technology, 5(3), 897–903. 10.1007/s11947-010-0363-z

[fsn31665-bib-0055] Mussatto, S. I. , Carneiro, L. M. , Silva, J. P. A. , Roberto, I. C. , & Teixeira, J. A. (2010). A study on chemical constituents and sugars extraction from spent coffee grounds. Carbohydrate Polymers, 83, 368–374. 10.1016/j.carbpol.2010.07.063

[fsn31665-bib-0056] Nakano, T. , Ikawa, N. , & Ozimek, L. (2003). Chemical composition of chicken eggshell and shell membranes. Poultry Science, 82, 510–554. 10.1093/ps/82.3.510 12705414

[fsn31665-bib-0057] Ogawa, M. , Portier, R. , Moody, M. , & Bell, J. (2004). Biochemical properties of bone and scale collagens isolated from the subtropical fish black drum (*Pogonia cromis*) and sheepshead seabream (*Archosargus probatocephalus*). Food Chemistry, 88, 495–501.

[fsn31665-bib-0059] Pandey, A. , Soccol, C. R. , Nigam, P. , Brand, D. , Mohan, R. , & Roussos, S. (2000). Biotechnological potential of coffee pulp and coffee husk for bioprocesses. Biochemical Engineering Journal, 6, 153–162. 10959086 10.1016/s1369-703x(00)00084-x

[fsn31665-bib-0060] Pantelić, M. M. , Dabić Zagorac, D. Č. , Davidović, S. M. , Todić, S. R. , Bešlić, Z. S. , Gašić, U. M. , … Natić, M. M. (2016). Identification and quantification of phenolic compounds in berry skin, pulp, and seeds in 13 grapevine varieties grown in Serbia. Food Chemistry, 211, 243–252. 10.1016/j.foodchem.2016.05.051 27283628

[fsn31665-bib-0061] Peschel, W. , Sánchez‐Rabaneda, F. , Diekmann, W. , Plescher, A. , Gartzía, I. , Jiménez, D. , … Codina, C. (2006). An industrial approach in the search of natural antioxidants from vegetable and fruit wastes. Food Chemistry, 97, 137–150. 10.1016/j.foodchem.2005.03.033

[fsn31665-bib-0062] Prado, A. C. P. , Silva, H. S. , Silveira, S. M. , Barreto, P. L. M. , Vieira, C. R. W. , Maraschin, M. , … Block, J. M. (2014). Effect of the extraction process on the phenolic compounds profile and the antioxidant and antimicrobial activity of extracts of pecan nut [*Carya illinoinensis* (Wangenh) C. Koch]. Industrial Crops and Products, 52, 552–561.

[fsn31665-bib-0063] Qiu, J. , Chen, L. , Zhu, Q. , Wang, D. , Wang, W. , Sun, X. , & Chemistry, X. L. (2012). Screening natural antioxidants in peanut shell using DPPH–HPLC–DAD–TOF/MS methods. Food Chemistry, 135, 2366–2371. 22980814 10.1016/j.foodchem.2012.07.042

[fsn31665-bib-0064] Raymond Chia, T. W. , & Dykes, G. A. (2010). Antimicrobial activity of crude epicarp and seed extracts from mature avocado fruit (*Persea americana*) of three cultivars. Pharmaceutical Biology, 48(7), 753–756. 10.3109/13880200903273922 20645772

[fsn31665-bib-0065] Ribeiro, S. M. R. , Barbosa, L. C. A. , Queiroz, J. H. , Knödler, M. , & Schieber, A. (2008). Phenolic compounds and antioxidant capacity of Brazilian mango (*Mangifera indica* L.) varieties. Food Chemistry, 110(3), 620–626. 10.1016/j.foodchem.2008.02.067

[fsn31665-bib-0066] Sabater, C. , Sabater, V. , Olano, A. , Montilla, A. , & Corzo, N. (2020). Ultrasound‐assisted extraction of pectin from artichoke by‐products. An artificial neural network approach to pectin characterisation. Food Hydrocolloids, 98, 105238 10.1016/j.foodhyd.2019.105238

[fsn31665-bib-0067] Salem, R. , & Abd El‐Ghany, M. (2012). Chemical and nutritional evaluation of different seed flours as novel sources of protein. World Journal of Dairy & Food Sciences, 7, 59–65.

[fsn31665-bib-0068] Sandhu, A. K. , & Gu, L. (2010). Antioxidant capacity, phenolic content, and profiling of phenolic compounds in the seeds, skin, and pulp of vitis rotundifolia (Muscadine Grapes) as determined by HPLC‐DAD‐ESI‐MSn. Journal of Agricultural and Food Chemistry, 58(8), 4681–4692. 10.1021/jf904211q 20334341

[fsn31665-bib-0069] Saura‐Calixto, F. , & Serrano, J. I. G. (2007). Intake and bioaccessibility of total polyphenols in a whole diet. Food Chemistry, 1001(2), 492–501.

[fsn31665-bib-0070] Schaafsma, A. , Van Doormaal, J. J. , Muskiet, F. A. J. , Hofstede, G. J. H. , Pakan, I. , Van Der Veer, E. , … Hofstede, F. A. J. (2018). Positive effects of a chicken eggshell powder‐enriched vitamin‐mineral supplement on femoral neck bone mineral density in healthy late post‐menopausal Dutch women. British Journal of Nutrition, 87(3), 267–275. 10.1079/BJN2001515 12064336

[fsn31665-bib-0071] Schieber, A. , Stintzing, F. C. , & Carle, R. (2001). By‐products of plant food processing as a source of functional compounds—recent developments. Trends in Food Science & Technology, 12(11), 401–413.

[fsn31665-bib-0072] Shahidi, F. , & Ambigaipalan, P. (2015). Phenolics and polyphenolics in foods, beverages and spices: Antioxidant activity and health effects – A review. Journal of Functional Foods, 18, 820–897. 10.1016/j.jff.2015.06.018

[fsn31665-bib-0073] Shenoy, D. , Pai, A. , Vikas, R. K. , Neeraja, H. S. , Deeksha, J. S. , Nayak, C. , & Rao, C. V. (2011). A study on bioethanol production from cashew apple pulp and coffee pulp waste. Biomass and Bioenergy, 35(10), 4107–4111. 10.1016/j.biombioe.2011.05.016

[fsn31665-bib-0074] Slatnar, A. , & Mikulic‐Petkovsek, M. F. S. (2015). Identification and quantification of phenolic compounds in kernels, oil and bagasse pellets of common walnut (*Juglans regia* L.). Food Research International, 67, 255–263. 10.1016/j.foodres.2014.08.009 30011716

[fsn31665-bib-0075] Soong, Y.‐Y. , & Barlow, P. J. (2004). Antioxidant activity and phenolic content of selected fruit seeds. Food Chemistry, 88(3), 411–417. 10.1016/j.foodchem.2004.02.003

[fsn31665-bib-0076] Sousa, C. , Gabriel, C. , Cerqueira, F. , Manso, M. C. , & Vinha, A. F. (2015). Coffee industrial waste as a natural source of bioactive compounds with antibacterial and antifungal activities. Retrieved from bdigital.ufp.pt

[fsn31665-bib-0077] Sousa De Brito, E. , De Araújo, M. C. P. , Alves, R. E. , Carkeet, C. , Clevidence, B. A. , & Novotny, J. A. (2007). Anthocyanins present in selected tropical fruits: Acerola, jambolão, jussara, and guajiru. Journal of Agricultural and Food Chemistry, 55(23), 9389–9394. 10.1021/jf0715020 17929888

[fsn31665-bib-0078] Srinivas Murthy, P. , Navya, P. N. , & Murthy Pushpa, S. (2013). Production, statistical optimization and application of endoglucanase from Rhizopus stolonifer utilizing coffee husk. Bioprocess and Biosystems Engineering, 36(8), 1115–1123. 10.1007/s00449-012-0865-3 23223909

[fsn31665-bib-0079] Stintzing, F. C. , & Carle, R. (2004). Functional properties of anthocyanins and betalains in plants, food, and in human nutrition. Trends in Food Science and Technology, 15(1), 19–38. 10.1016/j.tifs.2003.07.004

[fsn31665-bib-0080] Toldrá, F. , Aristoy, M.‐C. , Mora, L. , & Reig, M. (2012). Innovations in value‐addition of edible meat by‐products. Meat Science, 92, 290–296. 10.1016/j.meatsci.2012.04.004 22560456

[fsn31665-bib-0081] Tuchila, C. , Jianu, I. , Rujescu, C. , & Butur, M. (2008). Evaluation of the antimicrobial activity of some plant extracts used as food additives. Journal of Food Agriculture and Environment, 6(3&4), 68–70.

[fsn31665-bib-0082] Verma, L. R. , & Joshi, V. K. (2000). Postharvest technology of fruits and vegetables: Handling, processing, fermentation, and waste management. New Delhi, India: Indus Publishing Company .

[fsn31665-bib-0083] Vladić, J. , Ambrus, R. , Szabó‐Révész, P. , Vasića, V. , Cvejina, A. , Pavlić, B. , & Vidović, S. (2016). Recycling of filter tea industry by‐products: Production of *A. millefolium* powder using spray drying technique. Industrial Crops and Products, 80, 197–206.

[fsn31665-bib-0084] Wadhwa, M. , & Bakshi, M. P. S. (2013). Utilization of fruit and vegetable wastes as livestock feed and as substrates for generation of other value‐added products. Bangkok, Thailand: Food and Agriculture Organization.

[fsn31665-bib-0085] Xu, L. , Sheldon, B. , Larick, D. , & Carawan, R. (2002). Recovery and utilization of useful by‐products from egg processing wastewater by electrocoagulation. Poultry Science, 81(6), 785–792. 10.1093/ps/81.6.785 12079044

[fsn31665-bib-0086] Zhao, X. , Chen, J. , & Du, F. (2012). Potential use of peanut by‐products in food processing: A review. Journal of Food Science and Technology, 49, 521–529. 10.1007/s13197-011-0449-2 24082262 PMC3550843

[fsn31665-bib-0087] Zhu, F. , Du, B. , Zheng, L. , & Li, J. (2015). Advance on the bioactivity and potential applications of dietary fibre from grape pomace. Food Chemistry, 186, 207–212. 10.1016/j.foodchem.2014.07.057 25976812

[fsn31665-bib-0088] Zhu, Z. , Gavahian, M. , Barba, F. J. , Roselló‐Soto, E. , Bursać Kovačević, D. , Putnik, P. , & Denoya, G. I. (2020). Valorization of waste and by‐products from food industries through the use of innovative technologies In Agri‐food industry strategies for healthy diets and sustainability (pp. 249–266). London, UK: Elsevier 10.1016/b978-0-12-817226-1.00011-4

